# Therapeutic Outcomes of Combined Eyelid Hygiene, Intense Pulsed Light, and Meibomian Gland Expression in Meibomian Gland Dysfunction

**DOI:** 10.3390/jcm14238406

**Published:** 2025-11-27

**Authors:** Chien-Cheng Chien, Yu-Min Chang, Chang-Min Liang, Ke-Hung Chien, Ming-Cheng Tai, Ting-Yi Lin, Tzu-Heng Weng

**Affiliations:** 1 Department of Ophthalmology, Tri-Service General Hospital and School of Medicine, National Defense Medical University, Taipei 114202, Taiwan; hotjazzmichael@hotmail.com (C.-C.C.); m7886916@yahoo.com.tw (Y.-M.C.); doc30875@yahoo.com.tw (C.-M.L.); yred8530@gmail.com (K.-H.C.); mingtai1966@yahoo.com.tw (M.-C.T.); 2 Graduate Institute of Medical Sciences, National Defense Medical University, Taipei 11490, Taiwan

**Keywords:** dry eye disease, eyelid hygiene, intense pulsed light, meibomian gland dysfunction, meibomian gland expression

## Abstract

**Background/Objectives**: This study aimed to assess the effectiveness of a combination therapy comprising eyelid hygiene, intense pulsed light (IPL), and meibomian gland expression in patients with meibomian gland dysfunction (MGD). **Methods**: This retrospective study included MGD patients who completed at least three sessions of combination therapy administered at 4-week intervals, with a minimum follow-up period of 6 months. Ocular surface parameters were evaluated at baseline and at 1, 3, and 6 months after treatment initiation. Based on the clinical response following the initial three sessions, patients were categorized into either the standard treatment group (3 sessions) or the extended treatment group, who received three additional sessions of the same combination therapy. **Results**: A total of 107 patients (77 females; 30 males) were enrolled. 74 patients received standard treatment, and 33 received extended treatment. In the standard group, significant improvements compared with baseline were observed in the Ocular Surface Disease Index score, non-invasive tear break-up time, corneal staining, lid margin plugging, telangiectasia, and the meibomian gland expressibility score. Conversely, in the extended group, only the meibomian gland expressibility score showed significant improvements at the 3-month follow-up. **Conclusions**: The standard combination therapy resulted in significant and durable improvements in approximately 70% of MGD patients, with effects persisting for at least 3 months post-treatment. These findings support the clinical utility of this multimodal approach and highlight the need for biomarkers to predict treatment response.

## 1. Introduction

Dry eye disease (DED), alternatively termed dry eye syndrome or keratoconjunctivitis sicca, represents one of the most commonly encountered ocular disorders leading patients to seek medical evaluation. DED may affect individuals of any age, although it is more commonly observed in older adults and women. Clinical symptoms include ocular dryness, discomfort, irritation, foreign body sensation, and intermittent blurred vision [1,2].

According to the Tear Film and Ocular Surface Society Dry Eye Workshop II (TFOS DEWS II), DED is characterized as a complex, multifactorial condition affecting the ocular surface, marked by disruption of tear film homeostasis and accompanied by various ocular symptoms [3]. Key pathological features include tear film instability, increased tear osmolarity, inflammation and damage of the ocular surface, as well as neurosensory abnormalities contributing to disease development [3]. In the latest TFOS DEWS III report, the definition of DED has been further refined, emphasizing that it is a multifactorial and symptomatic disease, rather than a syndrome. The report also introduces a more detailed etiology-based subclassification, facilitating individualized diagnosis and management [4]. Similarly, the Asia Dry Eye Society (ADES) defines DED as “a multifactorial disease characterized by an unstable tear film causing a variety of symptoms and/or visual impairment, potentially accompanied by ocular surface damage.” [5] ADES broadly classifies DED into aqueous-deficient, decreased wettability, and evaporative subtypes [5]. Among these, evaporative dry eye is the most prevalent and is frequently associated with meibomian gland dysfunction (MGD), which is considered the leading cause of DED in clinical practice [6,7,8].

According to the International Workshop on Meibomian Gland Dysfunction, MGD is defined as a chronic, diffuse abnormality of the meibomian glands, characterized by terminal duct obstruction and/or qualitative and quantitative changes in glandular secretion [9]. This dysfunction leads to lipid layer deficiency, increased tear evaporation, tear film instability, ocular surface inflammation, and hyperosmolarity [10]. Consequently, meibomian gland dysfunction can disrupt both the biochemical and biophysical integrity of the tear film, resulting in ocular surface damage and dry eye symptoms. Epidemiological studies indicate that the estimated pooled prevalence of MGD is approximately 35.8%, with a higher susceptibility observed in men compared to women. The prevalence of MGD varies widely across populations, with rates reported between 21.2% and 71.0% [11]. Based on the level of meibum secretion, MGD can be classified into three main types: hyposecretory, obstructive, and hypersecretory. Aging is recognized as a major risk factor for the development of MGD. It can cause both structural and functional alterations in the glands, including reduced proliferation of acinar basal cells, increased keratinization, inflammatory cell infiltration, changes in neural innervation, glandular atrophy, and eventually gland dropout [12].

Diagnosis of MGD requires a comprehensive, multimodal approach, encompassing assessment of patient-reported symptoms, eyelid margin morphology, qualitative and quantitative changes in meibomian gland secretions, gland dropout, tear film lipid layer, tear volume, osmolarity and stability, ocular surface alterations, and inflammatory status [13,14]. Therapeutic strategies for MGD aim to alleviate chronic symptoms by improving meibum quality or enhancing meibum secretion, thereby restoring the balance between the lipid and aqueous layers of the tear film and stabilizing the ocular surface [15]. Treatment modalities include warm compresses, enhanced eyelid hygiene, antibiotics, corticosteroids, essential fatty acid supplementation, lipid-containing artificial tears, meibomian gland expression (MGX), intraductal meibomian gland probing, thermal pulsation, and intense pulsed light (IPL) therapy [16,17,18,19].

Among these treatment options, IPL has emerged as a promising non-pharmacological approach. IPL is a polychromatic, broad-spectrum light-emitting device that delivers filtered light pulses of varying durations to induce selective photothermal effects in target tissues [2,20]. Originally designed for dermatological purposes—including the treatment of telangiectasia, hypertrichosis, wrinkles, spots, and pigmented lesions, and other skin conditions—IPL was first adapted for ophthalmic use in 2015, specifically for the treatment of MGD [21,22,23]. Previous studies have demonstrated that IPL effectively improves both subjective symptoms and clinical signs in patients with MGD, including lipid layer grade, tear breakup time, meibum quality and expressibility, tear film lipid layer thickness, and overall optical quality [23,24,25]. Furthermore, combination therapy with IPL and MGX has also been shown to enhance symptom relief and improve meibomian gland function in this population [26,27,28,29]. Although the precise mechanisms underlying IPL’s therapeutic effects in MGD remain incompletely understood, proposed mechanisms include eradication of Demodex mites, a reduction in telangiectasia, liquefaction and softening of meibum, and modulation of inflammatory mediator secretion [30,31,32,33,34].

This study aims to evaluate the efficacy, onset, and durability of a combination therapy comprising eyelid hygiene, IPL, and MGX in patients with MGD. In addition, it seeks to identify clinical parameters that may serve as early indicators of therapeutic response and help differentiate treatment responders from non-responders.

## 2. Materials and Methods

### 2.1. Ethical Statement

This study was approved by the Institutional Review Board of Tri-Service General Hospital, Taipei, Taiwan (IRB No. A202205121; approval date: 24 July 2022). To protect patient privacy, all identifiable information extracted from the database was anonymized and encrypted prior to analysis. Given the retrospective design of the study and the use of de-identified data, the requirement for written informed consent was waived by the Institutional Review Board. All research procedures were conducted in accordance with the ethical standards set forth in the Declaration of Helsinki.

### 2.2. Patients

This retrospective study identified patients with MGD who presented to Tri-Service General Hospital between October 2019 and July 2020. Inclusion criteria were as follows: age ≥ 20 years; a diagnosis of MGD based on the presence of ocular symptoms, such as dryness, stickiness, foreign body sensation, burning, stinging, or photophobia persisting for at least three months; and at least one of the following clinical signs: (1) lid margin abnormalities (including plugging, telangiectasia, thickening, or irregularity), or (2) abnormal meibum expressibility or reduced meibum quality. Moreover, only patients who completed the standard combination therapy of eyelid hygiene, IPL, and MGX (three sessions), with or without an extended course (three additional sessions), and who completed six months of follow-up from the initial evaluation were included. Exclusion criteria included ocular allergies, active infections, contact lens wear, any history of ocular surgery within the preceding 6 months, and other systemic or ocular conditions that could potentially affect tear film production, tear dynamics, or ocular surface health. The detailed inclusion and exclusion criteria are summarized in Table 1.

### 2.3. Experimental Design

**Figure 1:**
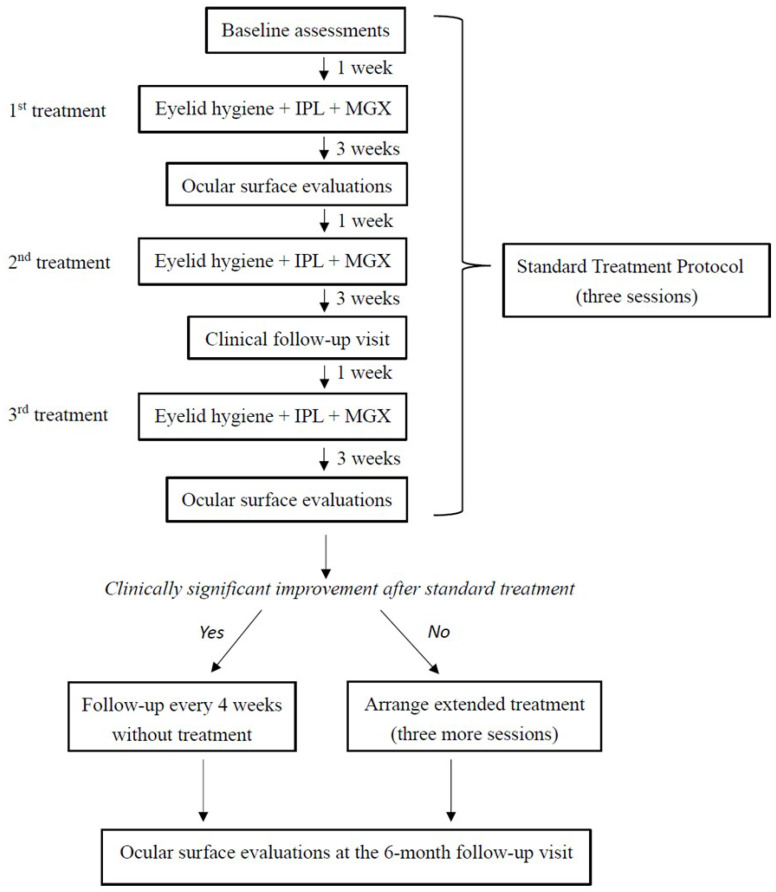
Treatment and follow-up protocol for combined treatment with eyelid hygiene, intense pulsed light, and meibomian gland expression.

This study aimed to evaluate the efficacy of combination therapy with eyelid hygiene, IPL, and MGX in patients diagnosed with MGD. Treatment sessions were administered at four-week intervals. A comprehensive ocular surface evaluation was performed for all participants one week prior to the initial treatment (baseline) and at follow-up visits conducted at 1, 3, and 6 months thereafter. Clinically significant improvement was defined as a concurrent improvement in both subjective symptoms, assessed by the Ocular Surface Disease Index (OSDI) score, and at least one objective clinical parameter, relative to baseline values, as observed at the 3-month follow-up visit. After completing the initial standard treatment course (three sessions), patients were stratified according to their treatment response. Those who exhibited clinically significant improvement were assigned to the standard treatment group and continued with routine follow-up until the 6-month visit without receiving additional interventions. Conversely, patients who did not exhibit such improvement (extended treatment group) underwent an additional three treatment sessions and were monitored until the 6-month follow-up. In other words, the standard treatment group received a total of three sessions, whereas the extended treatment group underwent six sessions in total (Figure 1).

### 2.4. Clinical Assessments

All participants underwent comprehensive ocular surface evaluations encompassing both subjective symptoms and objective clinical signs. Subjective symptoms were recorded using the validated OSDI questionnaire [35]. Objective assessments included noninvasive tear breakup time (NIBUT), tear meniscus height (TMH), and conjunctival redness score (scale 0–3) using the Oculus Keratograph 5M (Oculus Optikgeräte GmbH, Wetzlar, Germany). Corneal and conjunctival staining were graded using the Oxford Scheme, which scores staining from 0 to 5 based on the severity and extent of punctate staining, as described by Bron et al. (2003) [36]. Grade 0 (panel A) indicates no staining, whereas grades 1–4 correspond to increasing levels of punctate staining density and area, represented by reference panels B–E. Grade 5 denotes staining severity exceeding panel E (>E) of the Oxford Scheme [36]. Eyelid margin abnormalities of the lower eyelid, including plugging (scale 0–3), telangiectasia (scale 0–3), thickening (scale 0–2), and irregularity (scale 0–2) were assessed under slit-lamp [37]. The meibomian gland expressibility (MGE) score was determined by evaluating the eight central lower lid meibomian glands and meibum quality during expression (scale 0–3 per gland; total scale 0–24) [37].

### 2.5. Combined Treatment with Eyelid Hygiene, IPL, and MGX

At the outset, 0.5% proparacaine hydrochloride eye drops (Alcaine; Alcon, Inc., Geneva, Switzerland) were instilled to anesthetize the ocular surface. Prior to initiating combination therapy, eyelid cleansing was performed by moistening a washcloth with a tea tree oil-based cleanser and gently scrubbing the upper and lower eyelid margins. Subsequently, IPL treatment was administered using the M22 system (Lumenis, Yokneam, Israel), with the energy level adjusted to an appropriate setting (a range of 15 J/cm^2^). During the procedure, patients were instructed to keep their eyes closed, and protective corneal shields were placed to safeguard the ocular surface. A layer of cold ultrasonic gel was applied to the treatment area, followed by the delivery of approximately 12 light pulses to the lower eyelid region, covering a path from the left preauricular area, across the cheeks and nasal bridge, to the right preauricular area, with slight overlap between pulses and extending up to the base of the eyelashes. This sequence was repeated once. Thereafter, approximately eight pulses were delivered twice to the upper eyelid region, with the treated area extending down to the base of the eyelashes (also with slight overlap between pulses). Following eyelid hygiene and IPL treatment, MGX was performed on both the upper and lower eyelids using a meibomian gland compressor. Finally, a cold compress eyeshade was applied to the periocular area to alleviate post-procedural discomfort. All patients were also advised to continue routine ocular surface care at home, including regular warm compresses, eyelid cleansing, and blinking exercises.

### 2.6. Statistical Analysis

Continuous variables are presented as mean ± standard deviation (SD), ordinal variables as median with interquartile range (IQR), and categorical variables as number (percentage). The Kolmogorov–Smirnov test was used to assess the normality of all continuous variables. Between-group comparisons were performed using independent *t*-tests for normally distributed continuous variables, Wilcoxon rank-sum tests for ordinal variables, and the Chi-square test for categorical variables, as appropriate. Within-group changes in clinical parameters at each follow-up time point (1, 3, and 6 months) compared with baseline were analyzed as follows. For continuous variables satisfying the normality assumption, a one-way repeated-measures ANOVA was performed to assess overall differences across time points. When significant main effects were identified, pairwise comparisons with baseline were conducted using paired *t*-tests with Bonferroni adjustment. For non-normally distributed continuous variables or ordinal variables, the Friedman test was applied. When significant overall differences were detected, subsequent pairwise comparisons with baseline were performed using the Wilcoxon signed-rank test with Bonferroni correction (adjusted α = 0.0167). All statistical analyses were performed using SAS version 9.4 (SAS Institute, Cary, NC, USA), and a *p*-value < 0.05 was considered statistically significant, except when adjusted using the Bonferroni correction in the Wilcoxon signed-rank test.

## 3. Results

The baseline demographic and clinical characteristics of the enrolled patients are summarized in Table 2. A total of 107 patients with MGD were included in the study, comprising 77 females (72%) and 30 males (28%). The mean age was 64.3  ±  10.88 years (range, 21–85 years). In total, 74 patients received three sessions of combined treatment with eyelid hygiene, IPL, and MGX (standard treatment group), and 33 patients received six sessions (extended treatment group). All parameters at baseline showed no statistically significant differences between the two groups, except for the eyelid margin thickness score, which was significantly higher in the standard group compared to the extended group (*p*  =  0.031).

**Figure 2:**
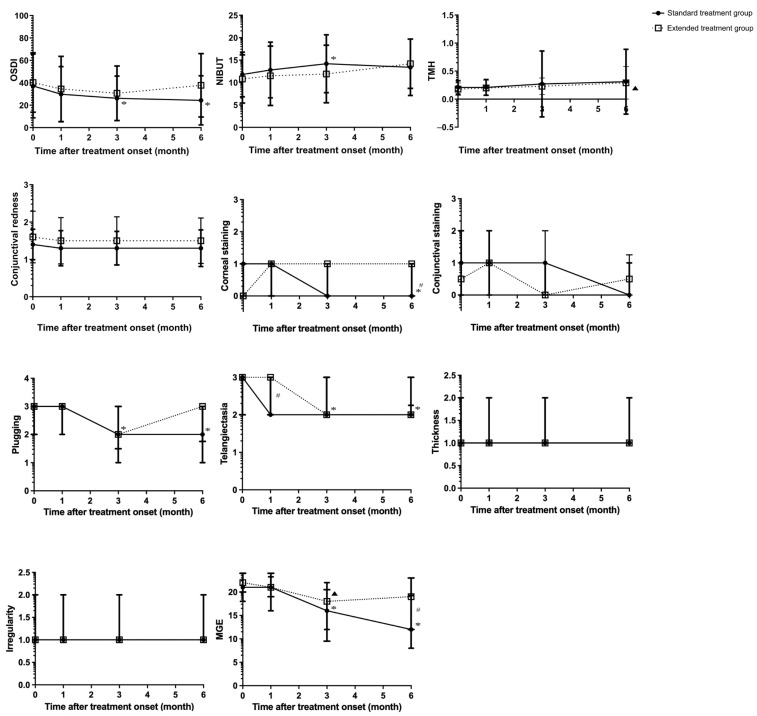
Changes in clinical parameters over time in the standard (three sessions) and extended (six sessions) treatment groups. * indicates an adjusted *p*-value showing a significant difference compared with the corresponding pretreatment value within the standard treatment group. ▲ indicates an adjusted *p*-value showing a significant difference compared with the corresponding pretreatment value within the extended treatment group. # indicates an adjusted *p*-value showing a significant difference between the standard and extended treatment groups at the specified time points.

Table 3 and Figure 2 summarize the comparative outcomes between the two groups, as well as the within-group changes from baseline at each follow-up time point. In the standard treatment group, the OSDI score showed a significant reduction from baseline at the 3-month follow-up, and this improvement was sustained at the 6-month assessments (*p* = 0.004, and *p* = 0.001, respectively; paired *t*-tests with Bonferroni adjustment). Similarly, NIBUT was significantly prolonged at the 3-month follow-up (*p* = 0.006; paired *t*-tests with Bonferroni adjustment), but this improvement was not maintained at 6 months. In contrast, although the extended treatment group exhibited trends toward improvement in both OSDI and NIBUT following treatment, these changes did not reach statistical significance at any follow-up time point. Notably, no significant differences were observed in TMH or conjunctival redness at any follow-up time point in either group. For ocular surface staining, the corneal staining score in the standard treatment group showed a significant reduction from baseline at the 6-month follow-up (*p* < 0.001; Wilcoxon signed-rank test with Bonferroni correction). Moreover, significant between-group differences favoring the standard group were observed at this time point (*p* = 0.014). In terms of lower eyelid margin abnormalities, both plugging and telangiectasia scores showed significant reductions from baseline in the standard treatment group at the 3- and 6-month follow-ups (*p* < 0.001 at both 3 and 6 months for plugging, and *p* = 0.003 and *p* < 0.001 for telangiectasia; Wilcoxon signed-rank test with Bonferroni correction). Conversely, the thickness and irregularity scores showed no significant changes from baseline at any follow-up time points in either group. In addition, significant between-group differences were observed only at baseline for the thickness score (*p* = 0.031) and at the 1-month follow-up for the telangiectasia score (*p* = 0.021). Regarding the MGE score, the standard treatment group showed significant decreases at the 3- and 6-month follow-ups, whereas the extended treatment group showed a significant decrease only at 3 months (all *p* < 0.001; Wilcoxon signed-rank test with Bonferroni correction). Additionally, a significant between-group difference favoring the standard treatment group was noted at the 6-month time point (*p* = 0.013).

To evaluate the potential influence of sex on treatment outcomes, the standard treatment group was further stratified by sex. As summarized in Supplementary Table S1, baseline demographic and clinical parameters did not differ significantly between male and female participants. Within-group analyses relative to baseline (Supplementary Table S2) demonstrated significant improvements in OSDI, NIBUT, corneal staining, plugging, telangiectasia, and MGE scores among female participants, findings that were generally consistent with those observed in the entire study population. In contrast, only lower eyelid telangiectasia showed a significant improvement in the male group following treatment. Moreover, between-group comparisons revealed significant sex differences in OSDI and corneal staining at the 6-month follow-up.

## 4. Discussion

This retrospective study enrolled 107 patients with MGD who underwent combination therapy consisting of eyelid hygiene, IPL, and MGX. Participants were divided into two groups according to their initial treatment response after three sessions of combined therapy (standard protocol). At baseline, there were no statistically significant differences in any of the evaluated parameters between the two groups, except for the lid margin thickness score, which was significantly higher in the standard treatment group. However, in this study, the lid margin thickness score showed no improvement in either group and, in fact, worsened in some cases following treatment. Based on the grading criteria, lid margin thickening was assessed on a scale from 0 to 2, depending on whether the thickening was localized or associated with diffuse rounding of the lid margin [37]. Due to the limited range of this scale and the subjective nature of the thickness evaluation, observer bias may have influenced this result. Therefore, this parameter should be interpreted with caution and may not reliably distinguish treatment responders from non-responders.

The parameters that showed statistically significant improvement in the standard treatment group were consistent with those commonly reported in previous IPL studies. According to a review article published in 2020, most clinical trials investigating IPL therapy demonstrated similar improvements across these parameters [2]. Of note, corneal staining and the MGE score were the only parameters to exhibit a statistically significant both intragroup and intergroup difference favoring the standard treatment group at the 6-month visit. Among the parameters of lid margin abnormalities, plugging and telangiectasia showed significant improvement following combined therapy. However, no meaningful improvements were observed in lid margin thickness or irregularity. This lack of response may reflect the fact that these latter features represent anatomical changes resulting from the long-term effects of chronic MGD. Therefore, meaningful improvement in thickness and irregularity may not be achievable after a relatively short treatment duration. The MGE score was the only parameter to show significant improvement in both groups at the 3-month follow-ups after three sessions of combined therapy. However, this effect persisted only in the standard treatment group at 6 months. This finding may be explained by the underlying pathophysiology of MGD, which is characterized by terminal duct obstruction and qualitative or quantitative alterations in glandular secretions [9]. These abnormalities can potentially be alleviated by IPL, which transfers heat through the periocular skin to melt the meibum and restore glandular function [23,33,38].

Three sessions of IPL combined with eyelid hygiene and MGX at 4-week intervals were used as the standard treatment protocol in this study, reflecting both our routine clinical practice and the methodology employed in several previous clinical trials [29,39]. Nevertheless, the optimal number of IPL sessions and the ideal intersession interval to achieve maximal therapeutic efficacy in patients with MGD remains an important clinical question [2]. A retrospective study by Toyos et al. found that patients with DED secondary to MGD who received five or more IPL sessions were 17.5 times more likely to show a statistically significant improvement in TBUT compared to those who received only one to three sessions. Additionally, nearly 90% of patients receiving four sessions reported clinical improvements in TBUT, meibum quality, lid margin condition, and patient satisfaction; however, the TBUT improvement was not statistically significant compared with the one-to-three-session group [23]. Lee et al., in their study evaluating the influence of varying numbers of treatment sessions for MGD, reported that IPL combined with MGX led to improvements in OSDI scores (after ≥3 sessions), meibomian gland expressibility (after ≥2 sessions), meibum quality (after all sessions), and tear breakup time (after all sessions) in patients with MGD [40]. Furthermore, a retrospective study involving 90 Asian adults with moderate-to-severe MGD compared the outcomes of three versus five IPL sessions. The authors concluded that increasing the number of sessions improved the overall treatment response rate but did not significantly enhance the magnitude of individual parameter improvement. They suggested that additional sessions may be unnecessary for patients who exhibit favorable responses to the standard treatment course [41].

In the subgroup analysis of the standard treatment group stratified by sex, a more pronounced improvement across multiple clinical parameters—including OSDI, NIBUT, corneal staining, plugging, and MGE scores—was observed in the female group, whereas only limited improvement was noted in the male group. Previous studies have demonstrated that sex- and gender-related differences significantly influence ocular surface homeostasis through the effects of hormones, sex chromosomes, sex-specific autosomal factors, epigenetics, as well as differences in care-seeking behaviors and health service utilization [42]. These biological and behavioral factors collectively affect lacrimal and meibomian gland function, corneal sensitivity, blinking behavior, immune regulation, and pain perception. Hormonal and genetic influences may further modulate inflammation, glandular aging, and tear film stability, contributing to the higher prevalence and symptom burden of DED observed in women [43]. These findings underscore the importance of integrating sex- and gender-based perspectives into the diagnosis, management, and therapeutic development for ocular surface disorders.

In the present study, although the extended treatment group showed modest improvements in several parameters following the initial three treatment sessions, only the MGE score reached statistical significance, and the therapeutic effects were not sustained despite additional sessions of combined therapy. In contrast, patients with MGD who received only the standard combined treatment exhibited statistically significant improvements in OSDI, NIBUT, lower lid plugging, telangiectasia, and the MGE scores at the 3-month follow-up. Importantly, most of these improvements were sustained and even further enhanced at the 6-month follow-up, despite the absence of additional treatment. This suggests that patients who respond favorably to the standard treatment protocol may maintain, and further improve, clinical benefits for at least three months without further IPL sessions. Despite these findings, the underlying reasons for the variability in treatment response remain unclear. No significant factors were identified to explain the differences between treatment responders and non-responders, and no clinical parameter reliably distinguished between the two groups. These findings suggest that individual variability in treatment response may be influenced by factors not captured by the current clinical measures, highlighting the need for further investigation into potential biomarkers or predictive indicators of treatment efficacy.

This study has several limitations: First, the relatively small sample size may have reduced the statistical power to define an optimal treatment protocol for MGD. Second, although baseline characteristics were generally comparable, patients were not stratified by disease severity, introducing potential heterogeneity in treatment response. Third, the follow-up period was relatively short, limiting the assessment of long-term effects. Fourth, adherence to recommended home care, including warm compresses, eyelid cleansing, and blinking exercises, was not monitored. Fifth, the study population included only Asian patients from Taiwan, which may limit generalizability to other ethnic groups. Sixth, the mechanisms by which IPL improves MGD remain incompletely understood. Finally, the retrospective design carries inherent limitations, such as potential selection bias and a lack of randomization. Prospective, randomized controlled trials with larger, more diverse populations are needed to confirm these findings and further clarify the therapeutic role of combined therapy in MGD.

## 5. Conclusions

In conclusion, this study demonstrates that the standard combination therapy of eyelid hygiene, IPL, and MGX produces clinically significant improvements in both subjective symptoms and objective clinical parameters in approximately 70% of patients with MGD, with these effects appearing more pronounced in female patients. Among those who responded, these benefits persisted for at least three months without additional treatment. These findings support the efficacy of the standard treatment protocol and underscore the need for further research to identify potential biomarkers or predictive indicators of treatment response.

## Figures and Tables

**Table 1:** Inclusion and exclusion criteria of the study.

Category	Criteria
Inclusion criteria	Age ≥ 20 years Diagnosis of MGD: - Ocular symptoms (dryness, stickiness, foreign body sensation, burning, stinging, or photophobia) persisting for at least three months - Plus one of the following clinical signs: (1) Lid margin abnormalities (plugging, telangiectasia, thickening, or irregularity) (2) Abnormal meibum expressibility or reduced meibum quality Completion of standard combination therapy (three sessions of eyelid hygiene, IPL, and MGX) with or without an extended course (three additional sessions) Completion of 6-month follow-up from the initial evaluation
Exclusion criteria	Ocular allergies Active ocular infections Contact lens wear History of ocular surgery within the previous 6 months Systemic or ocular conditions that could affect tear film production, tear dynamics, or ocular surface health (such as autoimmune diseases, endocrine disorders, or dermatological conditions)

**Table 2:** Baseline demographic characteristics of patients with meibomian gland dysfunction who received combined treatment with eyelid hygiene, intense pulsed light, and meibomian gland expression, categorized according to treatment response.

Parameter	Overall N = 107	Standard Treatment Group N = 74	Extended Treatment Group N = 33	*p*-Value
Sex				
Male	30 (28.0)	20 (27.0)	10 (30.3)	0.728
Female	77 (72.0)	54 (73.0)	23 (69.7)	
Age	64.3 ± 10.88 (21–85)	65.3 ± 9.17 (32–81)	62.0 ± 13.87 (21–85)	0.302
OSDI	38.1 ± 27.75 (0–100)	37.2 ± 28.42 (0–100)	40.3 ± 26.47 (0–100)	0.463
NIBUT	11.5 ± 5.10 (0–22.1)	11.8 ± 4.98 (2.9–22.1)	10.8 ± 5.38 (0–19.0)	0.339
TMH	0.20 ± 0.12 (0.06–0.75)	0.21 ± 0.12 (0.06–0.75)	0.18 ± 0.11 (0.06–0.62)	0.144
Conjunctival redness	14 ± 0.52 (0.4–2.9)	1.4 ± 0.41 (0.7–2.8)	1.6 ± 0.69 (0.4–2.9)	0.313
Corneal staining	1 (0–1)	1 (0–1)	0 (0–1)	0.196
Conjunctival staining	1 (0–1)	1 (0–2)	0.5 (0–1)	0.564
Plugging	3 (2–3)	3 (2–3)	3 (2–3)	0.497
Telangiectasia	3 (2–3)	3 (2–3)	3 (2–3)	0.752
Thickness	1 (1–2)	1 (1–2)	1 (1–1)	0.031 *
Irregularity	1 (1–2)	1 (1–2)	1 (1–2)	0.406
MGE	21 (19–24)	21 (18–24)	22 (20–24)	0.512

MGE, meibomian gland expressibility score; NIBUT, noninvasive tear breakup time; OSDI, Ocular Surface Disease Index; TMH, Tear meniscus height. Categorical variables are presented as number (percentage), continuous variables are expressed as mean ± standard deviation (range), and ordinal variables are summarized as median (interquartile range). * *p*-value < 0.05.

**Table 3:** Changes in clinical parameters in the standard treatment group (three sessions) and extended treatment group (six sessions) at baseline and at 1-, 3-, and 6-month follow-up visits after treatment initiation.

Parameter	Group	Baseline			1 Month After Treatment			3 Months After Treatment			6 Months After Treatment		
			*p*-Value †	Overall *p*-Value #		*p*-Value †	*p*-Value ‡		*p*-Value †	*p*-Value ‡		*p*-Value †	*p*-Value ‡
OSDI	Standard	37.2 ± 28.42	0.463	<0.001 *	29.9 ± 24.60	0.679	0.173	26.2 ± 19.81	0.714	0.004 §	24.4 ± 21.95	0.2857	0.001 §
	Extended	40.3 ± 26.47		0.291	34.5 ± 29.13		0.252	30.7 ± 24.38		0.53	37.8 ± 28.25		1.000
NIBUT	Standard	11.8 ± 4.98	0.339	0.008 *	12.8 ± 6.21	0.409	0.732	14.2 ± 6.46	0.302	0.006 §	13.4 ± 6.30	0.5970	0.260
	Extended	10.8 ± 5.38		0.077	11.5 ± 6.64		1.000	11.9 ± 6.42		1.000	14.2 ± 5.50		0.453
TMH	Standard	0.21 ± 0.12	0.144	0.192	0.21 ± 0.14	0.461	1.000	0.27 ± 0.59	0.466	1.000	0.31 ± 0.58	0.4477	0.654
	Extended	0.18 ± 0.11		0.137	0.20 ± 0.14		1.000	0.23 ± 0.15		1.000	0.29 ± 0.29		0.594
Conjunctival redness	Standard	1.4 ± 0.41	0.313	0.067	1.3 ± 0.47	0.945	1.000	1.3 ± 0.45	0.637	0.315	1.3 ± 0.49	0.429	0.103
	Extended	1.6 ± 0.69		0.131	1.5 ± 0.62		1.000	1.5 ± 0.64		0.282	1.5 ± 0.61		0.642
Corneal staining	Standard	1 (0–1)	0.196	<0.001 *	1 (0–1)	0.366	1.000	0 (0–1)	0.173	0.023	0 (0–1)	0.014 *	<0.001 §
	Extended	0 (0–1)		0.658	1 (0–1)		1.000	1 (0–1)		0.99	1 (0–1)		1.000
Conjunctival staining	Standard	1 (0–2)	0.564	0.568	1 (0–2)	0.964	1.000	1 (0–1)	0.795	1.000	0 (0–1)	0.591	0.434
	Extended	0.5 (0–1)		0.908	1 (0–1)		0.888	0 (0–2)		1.000	0.5 (0–1.25)		1.000
Plugging	Standard	3 (2–3)	0.497	<0.001 *	3 (2–3)	0.062	1.000	2 (1–3)	0.823	<0.001 §	2 (1–3)	0.092	<0.001§
	Extended	3 (2–3)		0.159	3 (3–3)		0.745	2 (1.5–3)		0.05	3 (1.75–3)		0.569
Telangiectasia	Standard	3 (2–3)	0.752	<0.001 *	2 (2–3)	0.021 *	0.443	2 (2–3)	0.311	0.003 §	2 (2–2.25)	0.124	<0.001 §
	Extended	3 (2–3)		0.073	3 (2–3)		1.000	2 (2–3)		0.178	2 (2–3)		0.319
Thickness	Standard	1 (1–2)	0.031 *	0.322	1 (1–2)	0.061	0.143	1 (1–2)	0.895	1.000	1 (1–2)	0.317	1.000
	Extended	1 (1–1)		0.039 *	1 (1–2)		0.25	1 (1–2)		0.066	1 (1–2)		0.038
Irregularity	Standard	1 (1–2)	0.406	0.308	1 (1–2)	0.572	0.182	1 (1–2)	0.713	1.000	1 (1–2)	0.583	1.000
	Extended	1 (1–2)		0.974	1 (1–2)		1.000	1 (1–2)		1.000	1 (1–2)		1.000
MGE	Standard	21 (18–24)	0.512	<0.001 *	21 (16–23.25)	0.574	0.055	16 (12–22)	0.672	<0.001 §	12 (8–19.25)	0.013 *	<0.001 §
	Extended	22 (20–24)		0.036 *	21 (19–24)		0.371	18 (9.5–20.5)		<0.001 §	19 (12–23)		0.041

MGE, meibomian gland expressibility score; NIBUT, noninvasive tear breakup time; OSDI, Ocular Surface Disease Index; TMH, Tear meniscus height. Continuous variables are expressed as mean ± standard deviation, and ordinal variables are summarized as median (interquartile range). # Overall *p* values were calculated using the Friedman test for ordinal data and one-way repeated-measures ANOVA for continuous data. † Indicates adjusted *p*-value for between-group comparisons (standard vs. extended treatment group) performed at each time point. ‡ Indicates adjusted *p*-value for within-group comparisons relative to baseline. * *p*-value < 0.05. § Statistically significant after Bonferroni correction for multiple pairwise comparisons within the same group.

## Data Availability

All data generated or analyzed during this study are included in this article. Further enquiries can be directed to the corresponding authors.

## Supplementary Materials

The following supporting information can be downloaded at https://www.mdpi.com/article/10.3390/jcm14238406/s1, Supplementary Table S1: Baseline demographic characteristics of patients in the standard and extended treatment groups, stratified by sex; Supplementary Table S2: Changes in clinical parameters in the standard treatment group at baseline and at 1-, 3-, and 6-month follow-up visits after treatment initiation, stratified by sex.

## Abbreviations

The following abbreviations are used in this manuscript:

ADES	Asia Dry Eye Society
DED	Dry Eye Disease
IPL	Intense Pulsed Light
MGD	Meibomian Gland Dysfunction
MGE	Meibomian Gland Expressibility
MGX	Meibomian Gland Expression
NIBUT	Noninvasive Tear Breakup Time
OSDI	Ocular Surface Disease Index
SAS	Statistical Analysis Software
TFOS DEWS II	Tear Film and Ocular Surface Society Dry Eye Workshop II
TMH	Tear Meniscus Height
