# Supplementary material for: Therapeutic Outcomes of Combined Eyelid Hygiene, Intense Pulsed Light, and Meibomian Gland Expression in Meibomian Gland Dysfunction
Source: J Clin Med. 2025 Nov 27;14(23):8406. doi: 10.3390/jcm14238406 (PMC12692845; doi:10.3390/jcm14238406)
Supplement: Supplementary file 1 [file jcm-14-08406-s001.zip › jcm-3965578-supplementary.pdf]

Supplementary Table S1. Baseline demographic characteristics of patients in the standard and extended treatment groups, stratified by sex.

**Parameter**	**Standard treatment group**		**P-value**	**Extended treatment group**		**P-value**
	Male	Female		Male	Female	
	**N**=20	**N**=54		**N**=10	N=23	
Age	66.95±7.19	64.69±9.79	0.281	62.2±16.12	61.87±13.17	0.928
OSDI	33.75±31.57	38.43±27.38	0.105	31.88±25.59	44.02±26.55	0.935
NIBUT	8.31±4.43	7.04±4.46	0.984	7.15±4.66	5.53±3.17	0.393
TMH	0.22±0.08	0.21±0.14	0.103	0.2±0.07	0.18±0.12	0.134
Conjunctival redness	1.52±0.48	1.34±0.38	0.565	1.79±0.64	1.46±0.71	0.698
Corneal staining	1 (0–1)	1 (0–1)	0.176	0 (0-1)	1 (0-1)	0.406
Conjunctival staining	0 (0–1)	1 (0–2)	0.051	0.5 (0-1.25)	1 (0-1)	0.985
Plugging	3 (2–3)	3 (2–3)	0.76	3 (2.75–3)	3 (2–3)	0.451
Telangiectasia	3 (2.25–3)	3 (2–3)	0.052	3 (2–3)	3 (2–3)	0.603
Thickness	1 (1–2)	1 (1–2)	0.884	1 (0.75-2)	1 (1-1)	0.524
Irregularity	1 (1–1.75)	1 (1–2)	1.000	1 (1–2)	1 (1–2)	0.686
MGE	20.5 (14.5–24)	21.5 (19.5–24)	0.211	22 (20-24)	20.5 (19.75-24)	0.499

MGE, meibomian gland expressibility score; NIBUT, noninvasive tear breakup time; OSDI, Ocular Surface Disease Index; TMH, Tear meniscus height

Categorical variables are presented as number (percentage), continuous variables are expressed as mean ± standard deviation (range), and ordinal variables are summarized as median (interquartile range).

Supplementary Table S2. Changes in clinical parameters in the standard treatment group at baseline and at 1-, 3-, and 6-month follow-up visits after treatment initiation, stratified by sex.

Parameter	Group	Baseline			1 month after treatment			3 months after treatment			6 months after treatment		
			P-value†	Overall *P*#		*P*-value†	*P*-value‡		*P*-value†	*P*-value‡		*P*-value†	*P*-value‡
OSDI	Male	33.75±31.57	0.105	0.034*	26.58±27.67	0.483	1.000	20±16.42	0.103	0.179	16.25±10.22	0.007*	0.065
	Female	38.43±27.38		0.008*	31.13±23.53		0.327	28.47±20.59		0.051	27.48±24.32		0.027*
NIBUT	Male	8.31±4.43	0.984	0.426	14.27±6.76	0.207	0.421	13.86±5.03	0.766	1.000	13.3±5.72	0.967	1.000
	Female	7.04±4.46		0.005*	12.21±5.97		1.000	14.37±6.95		0.015*	13.37±6.55		0.394
TMH	Male	0.22±0.08	0.103	0.078	0.22±0.08	0.775	1.000	0.26±0.17	0.894	1.000	0.32±0.18	0.939	0.283
	Female	0.21±0.14		0.325	0.21±0.15		1.000	0.28±0.69		1.000	0.31±0.69		1.000
Conjunctival redness	Male	1.52±0.48	0.565	0.039*	1.31±0.46	0.679	0.128	1.47±0.57	0.082	1.000	1.38±0.52	0.382	0.147
	Female	1.34±0.38		0.067	1.36±0.48		1.000	1.26±0.39		0.355	1.26±0.48		0.812
Corneal staining	Male	1 (0–1)	0.176	0.046*	0 (0-1)	0.072	1.000	1 (0-1)	0.725	1.000	0 (0-0)	0.048*	0.156
	Female	1 (0–1)		<0.001*	1 (0-1)		1.000	0 (0-1)		0.012§	0 (0-1)		0.001§
Conjunctival staining	Male	0 (0–1)	0.051	0.745	0 (0-1)	0.422	-	0 (0-1)	0.032*	-	0 (0-1)	0.084	-
	Female	1 (0–2)		0.745	1 (0-2)		-	1 (0-2)		-	0 (0-2)		-
Plugging	Male	3 (2–3)	0.76	0.041*	3 (2-3)	0.865	1.000	2 (2-3)	0.525	0.099	2 (1-3)	1.000	0.051
	Female	3 (2–3)		<0.001*	3 (2-3)		1.000	2 (1-3)		0.003§	2 (1-3)		0.003§
Telangiectasia	Male	3 (2.25–3)	0.052	0.009*	2 (2-3)	0.659	0.063	2 (2-3)	0.287	0.021	2 (2-3)	0.303	0.015§
	Female	3 (2–3)		0.002*	2 (2-3)		1.000	2 (2-3)		0.048	2 (1-2)		0.009§
Thickness	Male	1 (1–2)	0.884	0.678	1 (1-2)	0.645	-	1 (1-2)	0.217	-	1 (1-2)	0.797	-
	Female	1 (1–2)		0.249	1 (1-2)		-	1 (1-2)		-	1 (1-2)		-
Irregularity	Male	1 (1–1.75)	1.000	0.288	1 (1-2)	0.799	-	1 (1-2)	0.021*	-	1 (1-2)	0.897	-

	Female	1 (1–2)		0.350	1 (1-2)		-	1 (1-1.5)		-	1 (1-2)		-
MGE	Male	20.5 (14.5–24)	0.211	<0.001*	21 (14-24)	0.547	1.000	18 (16-22)	0.093	1.000	17 (8-22)	0.214	0.102
	Female	21.5 (19.5–24)		<0.001*	21 (16-23)		0.009§	16 (8.5-21.5)		<0.001§	12 (8-18)		<0.001§

MGE, meibomian gland expressibility score; NIBUT, noninvasive tear breakup time; OSDI, Ocular Surface Disease Index; TMH, Tear meniscus height.

Continuous variables are expressed as mean ± standard deviation, and ordinal variables are summarized as median (interquartile range).

# Overall p values were calculated using the Friedman test for ordinal data and one-way repeated-measures ANOVA for continuous data.

† Indicates adjusted P-value for between-group comparisons (standard vs. extended treatment group) performed at each time point.

‡ Indicates adjusted P-value for within-group comparisons relative to baseline.

* P-value<0.05.

§P-value < 0.0167, significant after Bonferroni correction for multiple pairwise comparisons within the same group.
